# Metabolic Pathways and Molecular Regulatory Mechanisms of Nervonic Acid Biosynthesis in *Malania oleifera*

**DOI:** 10.3390/ijms27125507

**Published:** 2026-06-18

**Authors:** Qijiang Xu, Chengyu Jiang, Mingyou Dong, Lusheng Liao, Guangfu Pang, Zhiyong Xing, Siyue Qi, Bo Zhou

**Affiliations:** 1 School of Medical Technology and Artificial Intelligence, Youjiang Medical University for Nationalities, Baise 533000, China; qijiangxu@126.com (Q.X.);; 2 Guangxi Database Construction and Application Engineering Research Center for Intracorporal Pharmacochemistry of TCM, Youjiang Medical University for Nationalities, Baise 533000, China; 3 College of Life Science, Northeast Forestry University, Harbin 150040, China

**Keywords:** *Malania oleifera*, nervonic acid, metabolic pathway, fatty acid elongation, Δ15 desaturase, gene family analysis, transcriptional regulation, multi-omics, metabolic engineering

## Abstract

Nervonic acid (NA, C24:1 Δ15) is a vital extra-long-chain monounsaturated fatty acid essential for neural development, myelin sheath formation, and neurological health. As the most abundant natural source of NA, *Malania oleifera* Chun & S.K.Lee has become a key model for studying NA biosynthesis and regulation. This review systematically summarizes the metabolic pathways of nervonic acid biosynthesis in *M. oleifera*, including plastidial *de novo* fatty acid synthesis, endoplasmic reticulum (ER)-based very-long-chain fatty acid elongation, and Δ15 desaturation. We focus on the catalytic mechanisms and rate-limiting roles of the elongase complex (KCS, KCR, HCD, ECR) and Δ15 desaturase. Additionally, we integrate recent multi-omics data to analyze key enzyme KCS gene families, their phylogenetic relationships, and syntenic distribution patterns. Furthermore, transcriptional regulatory networks (MYB, bZIP, WRI1, ABI3, FUS3) and epigenetic regulation underlying NA accumulation are also discussed. Finally, we highlight advances, challenges, and prospects in metabolic engineering and synthetic biology for sustainable NA production. This review provides a theoretical basis for the conservation, molecular breeding, and biotechnological utilization of *M. oleifera*.

## 1. Introduction

Nervonic acid (NA, C24:1 Δ15) is a very long-chain monounsaturated fatty acid concentrated mainly in the mammalian central nervous system (CNS). There, it serves as a cornerstone structural element of the myelin sheath and the biological membranes enveloping brain neurons. Its unique structural and functional traits are crucial for maintaining neuronal membrane integrity and proper function, rendering it indispensable for both neurodevelopment and repair. Deficiencies or imbalances in NA levels have been implicated in a spectrum of neurodegenerative and demyelinating diseases, including Alzheimer’s disease, Parkinson’s disease, and schizophrenia, highlighting its potential as a therapeutic target and biomarker for neurological health [1,2,3]. Studies in vitro have demonstrated that NA can mitigate neuronal injury caused by oxidative stress, strengthen internal antioxidant defenses, and sustain cell survival under neurotoxic conditions [4]. Moreover, NA exhibits anti-inflammatory effects by modulating proinflammatory signaling pathways and metabolic networks. In animal models of Parkinson’s disease, NA successfully reduced both hepatic inflammation and systemic inflammatory responses [5]. These research results show that NA can serve as a promising bioactive lipid with significant implications for the prevention and management of neurodegenerative disorders.

**Figure 1:**
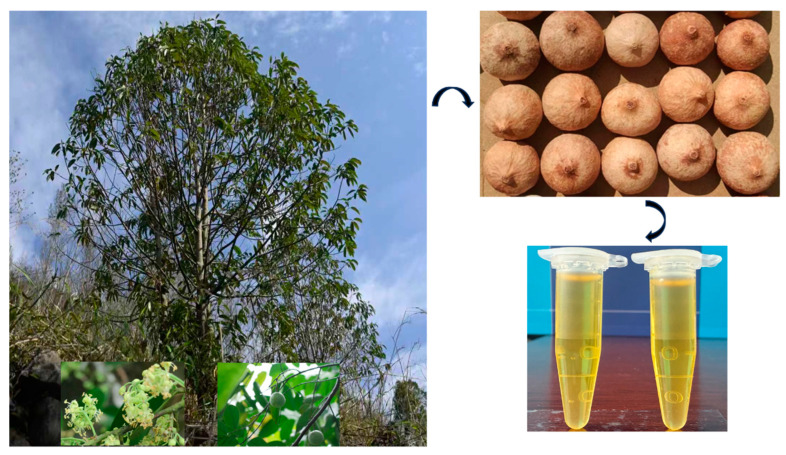
Wild-growing *M. oleifera* plants in their native habitat, harvested fruit, and seed oil content visualization.

Despite its key clinical and biological value, NA’s scarce, low-abundance natural sources hinder sustainable, large-scale production. Among known NA-rich plants, the rare endemic woody oilseed *Malania oleifera* Chun & S.K.Lee (*Olacaceae*) stands out with its ultra-high seed oil NA content as an ideal model for NA biosynthesis studies and biotechnological development [6]. However, the wild populations of *M. oleifera* (garlic-fruit tree) are endangered, and the species exhibits a prolonged growth cycle, which restricts direct agricultural utilization and large-scale harvesting (Figure 1). These limitations necessitate a comprehensive understanding of the metabolic pathways and molecular regulatory mechanisms governing NA biosynthesis in *M. oleifera* to enable alternative production strategies, such as metabolic engineering and synthetic biology approaches. Recent advances in high-throughput omics technologies, including genome sequencing, transcriptomics, and lipidomics, have facilitated the identification of key enzymes and regulatory factors involved in NA biosynthesis, providing a foundation for elucidating the complex metabolic networks in this species [6].

NA biosynthesis involves the elongation and desaturation of fatty acid precursors, catalyzed by a suite of enzymes including β-ketoacyl-CoA synthases (KCSs), β-ketoacyl-CoA reductases (KCRs), 3-hydroxyacyl-CoA dehydratases (HCDs), and enoyl-CoA reductases (ECRs). In *M. oleifera*, genomic analyses have revealed multiple candidate genes encoding these enzymes, with expression profiles correlating with seed development stages and NA accumulation, suggesting their pivotal roles in NA biosynthesis [6]. Complementary studies in engineered *Yarrowia lipolytica* and other microbial systems have demonstrated the feasibility of reconstructing orthogonal NA biosynthesis pathways via the co-expression of plant-derived elongases and desaturases, paired with metabolic engineering strategies to boost precursor supply and lipid accumulation for high titers of NA-enriched oils [7]. These findings highlight the potential of integrating plant and microbial platforms for sustainable NA production to bypass the ecological and agronomic constraints of *M. oleifera* cultivation.

Furthermore, the regulation of NA biosynthesis encompasses transcriptional and post-transcriptional mechanisms coordinating enzyme expression and activity in response to developmental cues and environmental factors. The elucidation of these molecular controls is critical for optimizing NA yield and tailoring fatty acid profiles in both native and heterologous systems. Advances in synthetic biology and metabolic engineering provide powerful tools to manipulate these regulatory circuits, enabling the design of customized biosynthetic pathways with enhanced efficiency and specificity [7,8] These approaches fit industrial biotechnology’s core goals of creating bio-based high-value lipid production systems to drive the bioeconomy and sustainable resource utilization [8].

Previous reviews broadly cover NA functions, biosynthesis, and applications across systems, but lack species-specific insights into its extreme accumulation in *M. oleifera*. To fill this gap, this review systematically consolidates current knowledge on the NA metabolic pathways and rate-limiting enzymes (KCS, FAD) in *M. oleifera*, and integrates recent multi-omics data to refine its molecular regulatory network. By highlighting recent progress and outlining future directions, it further proposes targeted metabolic engineering strategies based on the species’ unique advantages and bottlenecks. Ultimately, this work provides a focused, updated framework for both mechanistic research and the biotechnological exploitation of high-NA germplasm.

## 2. Biological Functions of Nervonic Acid and Resource Value of *Malania oleifera*

### 2.1. The Central Role of Nervonic Acid in the Nervous System

Nervonic acid (NA, ω-9 C24:1) is a very long-chain monounsaturated fatty acid that exists predominantly in central nervous system (CNS) sphingolipids, including sphingomyelin and cerebrosides, as well as in glycerophospholipids [9]. These lipid species are fundamental building blocks of neuronal cell membranes, where NA makes a major contribution to preserving membrane fluidity, structural stability, and the normal operation of signal transduction pathways. The unique biophysical traits of NA-containing lipids enable the formation of the dense, stable myelin sheaths that insulate axons and support the rapid saltatory conduction of nerve impulses. During the myelination process, NA acts as a direct precursor for the synthesis of ceramides and more complex sphingolipids, directly shaping the structural integrity and functional performance of the myelin sheath. Disruptions to NA metabolism or insufficient supply can compromise myelin stability, leading to impaired neural conductivity and subsequent neurological dysfunction [10].

Beyond structural roles, NA exhibits neuroprotective properties through its antioxidant and anti-inflammatory effects. Studies in vitro using neuronal cell models show that pre-treatment with NA significantly boosts cell survival by activating endogenous antioxidant defense pathways [11]. As a result, NA reduces levels of lipid peroxidation markers such as malondialdehyde, mitigating the oxidative damage that drives the progression of Parkinson’s and Alzheimer’s disease (AD) [4].

Clinical studies and animal model experiments further validate the therapeutic potential of NA supplementation for neurodegenerative and demyelinating disorders. In AD rat models, NA administration reversed cognitive deficits and reduced neuronal damage, likely through its ability to reshape gut microbiota composition and modulate metabolic pathways related to short-chain fatty acids and sphingolipid metabolism. Additionally, NA regulates key metabolic enzymes and pathways involved in linoleic acid, α-linolenic acid, and arachidonic acid metabolism, all of which are integral to sustaining neuronal health and function [3].

The research into NA’s role in remyelination and CNS regeneration shows that supplementation with natural lipids, including NA-rich fish oil, enhances the differentiation and maturation of oligodendrocyte precursor cells (OPCs) into fully functional, myelinating oligodendrocytes [12]. In mature oligodendrocytes, increased NA levels are paired with a higher synthesis of key myelin proteins: myelin basic protein (MBP), myelin oligodendrocyte glycoprotein (MOG), and proteolipid protein (PLP), directly demonstrating NA’s essential contribution to myelin repair mechanisms [13]. Fish oil supplementation also strengthens the integrity of BBB tight junctions, which further supports CNS homeostasis and improves remyelination outcomes [14]. These findings make a strong case for including NA in dietary strategies to support CNS regeneration and functional recovery following demyelinating injury.

Taken together, these findings establish that NA is irreplaceable for maintaining the structural and functional integrity of the nervous system. As a core lipid component of myelin and neuronal membranes, it is essential to preserving membrane fluidity, stability, and efficient signal transduction.

### 2.2. Uniqueness and Endangered Status of *Malania oleifera* as a Nervonic Acid-Enriched Plant

*Malania oleifera*, the garlic fruit tree, is a botanical resource of unparalleled value due to the extraordinary NA content of its seed oil, which ranges from 55% to 67% [15]. As a very long-chain monounsaturated fatty acid critical to neurological health, NA has highly promising applications in the pharmaceutical and nutraceutical industries. The exceptional accumulation of NA in *M. oleifera* seeds reflects a unique metabolic specialization within the species, and highlights its potential as a sustainable source for industrial NA extraction and therapeutic development [16]. Unfortunately, the natural distribution of *M. oleifera* is extremely restricted, limited almost exclusively to the karst landscapes of southwest China’s Yunnan and Guangxi Provinces [17].

Overharvesting and widespread habitat destruction have caused a dramatic collapse in wild populations of *M. oleifera*. Climate change presents an additional and urgent threat. In response to these challenges, conservation and sustainable utilization efforts for *M. oleifera* have increasingly focused on ex situ conservation strategies, most notably the development of efficient in vitro propagation protocols. Recent breakthroughs have achieved successful indirect somatic embryogenesis via callus induction from seedling explants, overcoming long-standing barriers to *M. oleifera* tissue culture such as phenolic exudation from explants. The use of antioxidant pretreatments and optimized carbon sources has drastically improved the callus induction rates and somatic embryo maturation, enabling the production of viable plantlets that can be acclimatized and transferred to greenhouse conditions. This advance in tissue culture technology not only supports the mass propagation of *M. oleifera* for population restoration and commercial cultivation, but also provides a platform for biochemical studies and genetic improvement to enhance NA yield [18].

Genomic insights provide a powerful tool to identify priority populations for genetic rescue and to design adaptive management strategies that account for future climate impacts. Meanwhile, biotechnological advances in propagation and cultivation can reduce harvesting pressure on wild populations and enable the sustainable use of this valuable resource. Protecting *M. oleifera* therefore requires a multifaceted strategy that unites habitat preservation, genetic conservation, and biotechnological innovation to ensure the survival and ongoing availability of this unmatched NA-rich plant species [16,18].

## 3. Core Metabolic Pathways for Nervonic Acid Biosynthesis in *Malania oleifera*

### 3.1. De Novo Fatty Acid Synthesis and the Production of Palmitic Acid

**Figure 2:**
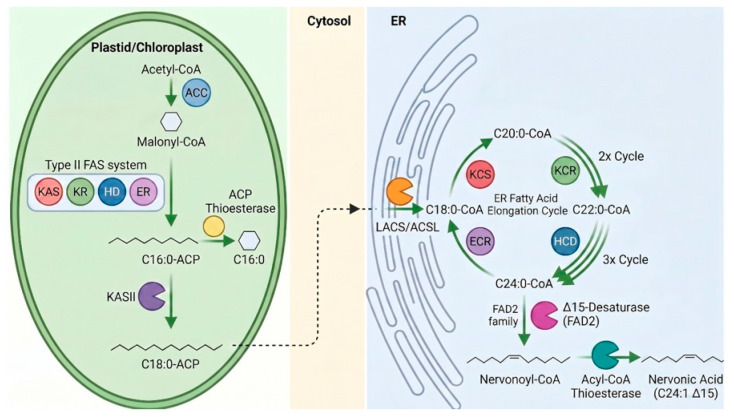
Compartmentalized biosynthetic pathway of nervonic acid (C24:1 Δ15) in *Malania oleifera*.

*De novo* fatty acid synthesis is a foundational metabolic pathway that occurs primarily in plant plastids, where acetyl-CoA is converted into saturated fatty acids, most commonly 16 or 18 carbons in length. The process is driven by a sequential series of enzymatic reactions catalyzed by acetyl-CoA carboxylase (ACC) and the fatty acid synthase (FAS) complex. The first committed step is catalyzed by ACC, which carboxylates acetyl-CoA to form malonyl-CoA, the two-carbon donor for subsequent fatty acid chain elongation. The FAS complex then adds malonyl-CoA units to the growing acyl chain in a cyclic manner, with acyl carrier protein (ACP) acting as a molecular shuttle that binds and transports reaction intermediates. The primary end product of this pathway is palmitic acid (C16:0), a saturated fatty acid that serves as the essential substrate for the downstream elongation and desaturation reactions that produce longer-chain and unsaturated fatty acids (Figure 2). Palmitic acid’s central role in lipid metabolism is reflected in its high abundance in plant tissues, and its function as a precursor for both complex structural lipids and bioactive signaling molecules.

NA biosynthesis occurs in two subcellular compartments. In the plastid, acetyl-CoA is carboxylated to malonyl-CoA by acetyl-CoA carboxylase (ACC), and the fatty acid synthase (FAS) complex produces C18:0 precursors. In the endoplasmic reticulum (ER), the four-step elongase complex (KCS, KCR, HCD, ECR) catalyzes iterative elongation to C24:0, with KCS as the rate-limiting enzyme. Finally, a specialized Δ15 desaturase introduces a cis-double bond to form nervonic acid (C24:1 Δ15).

In *M. oleifera*, the efficiency and metabolic flux of the de novo fatty acid synthesis pathway directly determine the availability of the initial carbon chains required for NA biosynthesis [19,20]. As a very long-chain monounsaturated fatty acid, NA can only be produced through the elongation of substrates derived from palmitic acid. The activity of core enzymes such as FAS and ACP directly shapes intracellular palmitic acid levels, and thus the overall capacity for NA production [21,22,23]. Studies across diverse organisms have demonstrated that modulating FAS activity causes significant shifts in palmitic acid accumulation [24,25].

In addition to enzymatic regulation, environmental factors including temperature and nutrient availability can alter fatty acid synthesis dynamics. Low-temperature stress in plants has been shown to shift fatty acid profiles and alter the expression of biosynthetic genes, leading to changes in palmitic acid content [26]. Such environmental modulation is likely relevant to *M. oleifera*, where adaptive shifts in fatty acid metabolism may optimize NA biosynthesis under fluctuating growth conditions.

### 3.2. Mechanism of Very Long-Chain Fatty Acid Elongation: Fatty Acid Elongase Complex

Nervonic acid (NA, C24:1 monounsaturated VLCFA) biosynthesis relies almost entirely on the ER-localized fatty acid elongase complex, which mediates sequential two-carbon unit additions to fatty acyl-CoA substrates for chain elongation to the C24 length required for NA. The complex comprises four core enzymes, each catalyzing a specific step of the four-stage cycle: β-ketoacyl-CoA generated by KCS is reduced to β-hydroxyacyl-CoA by KCR, then dehydrated to trans-2-enoyl-CoA by HCD, and finally reduced to the fully elongated C20 acyl-CoA by ECR. Two additional rounds of the KCS/KCR/HCD/ECR cycle extend C20:0-CoA to C24:0-CoA, and then C24:0 is catalyzed by Δ15 desaturase to undergo desaturation reaction to generate nervonic acid (C24:1) (Figure 2). Additionally, C18:0-acyl-CoA, C20:0-acyl-CoA, and C22:0-acyl-CoA are also sequentially desaturated by Δ9, Δ11, Δ13, and Δ15 desaturases to generate C18:1-acyl-CoA, C20:1-acyl-CoA, and C22:1-acyl-CoA, respectively. Structural studies show that TECR forms a stable complex with HCD [27]. In *Malania oleifera*, the specific isoform MoKCS is the key rate-limiting enzyme, in which malonyl-CoA (the two-carbon donor) condenses with acyl-CoA to form β-ketoacyl-CoA. The high substrate specificity for C22 monounsaturated fatty acids and catalytic efficiency are one of the key potential reasons for the high NA accumulation in *M. oleifera* seeds, making NA the dominant VLCFA in its seed oil [27].

Plant fatty acid elongase complexes possess unique functional characteristics distinct from their mammalian homologs. For instance, the plant β-ketoacyl-CoA synthase CER6 (KCS6) forms a heterotetrameric complex with the BAHD acyltransferase GL2, which remodels CER6’s substrate tunnel into a continuous hydrophobic channel, altering its substrate specificity to enable the elongation of acyl chains over 28 carbons for ultra-long fatty acid synthesis [28]. Though this mechanism has only been characterized in maize and Arabidopsis [29,30], it exemplifies the structural complexity and substrate specificity modulation of plant very-long-chain fatty acid (VLCFA) elongase complexes [28]. In cotton (Gossypium hirsutum), VLCFA biosynthesis (including nervonic acid (NA) precursor production) is tightly regulated by transcription factors GhBZR3 and GhBES1.4. These factors integrate brassinosteroid signals to modulate the expression of VLCFA elongation-related *KCS* genes (e.g., *GhKCS10*, *GhKCS13*), with specific binding motifs in the promoters of these *KCS* genes enabling the precise transcriptional control of elongase activity, thereby regulating fiber elongation via VLCFA production rate modulation [31,32].

Protein–protein interactions are pivotal for efficient plant VLCFA synthesis. Arabidopsis studies have identified homo- and hetero-oligomeric interactions between KCS isoforms, as well as between KCS and other elongase components (KCR1, PAS2, ECR) [33]. Specific amino acid residues in KCS9 are critical for such interactions and for its catalytic function in synthesizing C24 VLCFAs from C22 substrates. These interactions stabilize the elongase complex and facilitate inter-enzyme substrate channeling during the elongation cycle [33]. The molecular mechanisms of the function and regulation of the elongase complex in *M. oleifera* lay a foundation for future metabolic engineering strategies to enhance NA production [27,28,31,32,34,35].

### 3.3. Desaturation Step: The Key Role of ω-9 Desaturases

The formation of nervonic acid (NA, C24:1 Δ15) relies on a critical desaturation step. Specifically, a Δ15 desaturase introduces a cis double bond at the 15th carbon of C24 fatty acid chains. Unlike general fatty acid desaturases, which typically act on common positions such as Δ9, this specific Δ15 desaturation converts saturated or polyunsaturated C24 precursors into the bioactive monounsaturated NA, a structural feature that underpins its physiological and industrial significance [8,36].

Genomic and transcriptomic analyses of *Malania oleifera* (1.5 Gb genome, 13 pseudo-chromosomes) have identified efficient desaturase candidates from the FAD2 and FAD3 families in the species [16]. FAD2 and FAD3 serve as the principal enzymes mediating Δ12 and Δ15 desaturation within plant systems [37]. Research on systematic metabolic engineering for nervonic acid production identified and utilized the *M*. *oleifera* desaturase gene *MaOLE2*. This enzyme exhibits confirmed Δ15 desaturase activity, converting C24:0 (lignoceric acid) to C24:1 Δ15 (nervonic acid), thereby demonstrating the definitive presence of specific enzymes catalyzing ultra-long-chain fatty acid Δ15 desaturation in *M. oleifera* [36]. Nonetheless, the functions of *M. oleifera* Δ15 desaturases specific to C24 substrates demand further in vivo and in vitro experimental validation.

The expression of *M. oleifera* desaturase genes in developing seeds is tightly correlated with NA accumulation dynamics, indicating transcriptional regulation modulates desaturase availability and activity during seed maturation. This synchronization ensures that the desaturation step matches the physiological demand for NA, which is primarily stored in seed oil. The ω-9 desaturation step catalyzed by FAD2/FAD3-derived Δ15 desaturases is indispensable for NA biosynthesis in *M. oleifera* seeds.

## 4. Key Enzyme Gene Families Regulating Nervonic Acid Biosynthesis

### 4.1. Functional Traits and Divergence of the β-Ketoacyl-CoA Synthase Gene Family

The β-ketoacyl-CoA synthase (KCS) gene family coregulates plant very long-chain fatty acid (VLCFA) biosynthesis and produces precursors for nervonic acid (NA) in *Malania oleifera* [38,39]. These *KCS* member genes show tissue-specific (fruit, seed, leaf) and seed development stage-dependent expression, reflecting a refined regulatory system matching enzymatic activity to tissue and growth metabolic demands. The studies in *M. oleifera* confirm only two KCS isoforms, FAE-like members, that have high specificity and efficiency for C22:0/C22:1 and C24:0/C24:1 elongation [38]. As NA biosynthesis’s major drivers, these key isoforms catalyze the rate-limiting condensation reaction for C24 chain formation. Their functional specialization reveals profound KCS family divergence: some undertake housekeeping in general VLCFA synthesis, while others evolve to mediate specific elongation for high NA accumulation.

**Figure 3:**
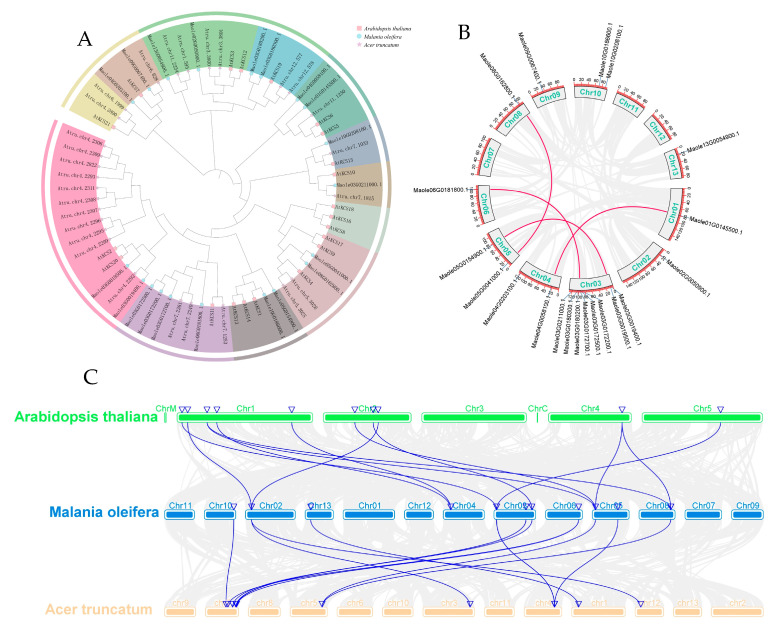
The phylogenetic tree and distribution of *KCS* genes based on protein sequences of *Arabidopsis*, *Acer truncatum*, and *M. oleifera*. (**A**) Phylogenetic tree of the *KCS* gene family, showing 12 clades in cluster analysis. (**B**) The intraspecific collinearity analysis of the *KCS* gene family. (**C**) The interspecific synteny analysis of the *KCS* gene family. Data analysis and visualization using MEGA12 [43] and TB-Tools [44].

Comparative genomic and phylogenetic analyses of *Arabidopsis* and *Acer truncatum KCS* genes provide evolutionary insights into the conservation and diversification of the KCS family in *M. oleifera* (Figure 3). Phylogenetic analysis showed that Maole03G0019400.1 and Maole03G0019500.1 (orthologous to AtKCS2 and AtKCS20) cluster with Atru.chr4.2307, Atru.chr4.2308, and Atru.chr4.2311. The expression profiles of *KCS* genes in *A. truncatum* indicate that these clustered genes likely share conserved roles in NA synthesis during seed development [39]. In the other two classes (purple and pink cluster in Figure 3A), Maole06G0181800, Maole03G0172700, Maole03G0172500, and Maole03G0172200 as well as Maole068G0162800 and Maole05G0041000, identified as orthologs of *Arabidopsis* AtKCS11 and AtKCS4, respectively, were also reported to likely be responsible for NA biosynthesis [6]. Intraspecific collinearity analysis revealed that eight of the twenty *KCS* genes in *M. oleifera*, distributed across different chromosomes, exhibited syntenic relationships. This pattern strongly suggests that these genes originated from ancient whole-genome duplication (WGD) or segmental duplication events, rather than tandem duplications. The retention of these syntenic KCS paralogs implies that they may have experienced purifying selection, potentially contributing to functional redundancy or sub-functionalization in very-long-chain fatty acid (VLCFA) biosynthesis during seed development (Figure 3B). Interspecific synteny analysis demonstrated highly conserved collinearity of the KCS gene family across the *M. oleifera*, *Arabidopsis*, and *A. truncatum* species. Specifically, 6 and 10 *KCS* genes in *M. oleifera* were syntenic with 10 and 9 genes in *Arabidopsis* and *A. truncatum*, respectively. The identification of 5 collinear genes shared across all three species suggests a common ancestral origin and strong evolutionary conservation of these specific KCS members (Figure 3C). Phylogenetic analysis and the uneven chromosomal distribution of *KCS* genes suggest lineage-specific expansion and functional specialization within this gene family [40]. In *M. oleifera*, 20 KCSs, 4 KCRs, 1 HCD, and 1 ECR were identified as candidate genes involved in NA biosynthesis in developing seeds [6]. This evolutionary pattern supports the hypothesis that *M. oleifera KCS* genes have undergone similar lineage-specific diversification to adapt to the species’ unique metabolic requirement for high NA accumulation. Additionally, maize abiotic stress analyses show that certain *KCS* genes (e.g., *ZmKCS17*) are significantly downregulated under stress, suggesting that environmental conditions may modulate VLCFA biosynthesis by regulating *KCS* gene expression [41,42]. These gene resources provide critical targets for future metabolic engineering and synthetic biology applications. Genomic and transcriptomic advances lay the groundwork for the biotechnological development of sustainable NA production [6].

### 4.2. Diversity of Fatty Acid Desaturase Genes and Their Substrate Specificity

Fatty acid desaturases (FADs) make up a diverse family of enzymes that catalyze the insertion of double bonds into fatty acyl chains, a critical step in the production of all unsaturated fatty acids. Heterologous expression systems can be used to confirm the desaturation activity and substrate specificity of FAD enzymes from diverse species. For example, front-end desaturases from annelids were characterized via heterologous expression in yeast, revealing their Δ5 and Δ6Δ8 desaturase regioselectivity, which is essential for long-chain PUFA biosynthesis [45]. Similarly, the Δ15 desaturase from cyanobacteria was shown to count double bond positions from the carboxyl end of the fatty acid chain, and to require specific substrate configurations for full activity [46].

Co-expression analyses have also revealed that certain FAD genes are co-regulated with elongase genes such as *KCS*, which catalyzes fatty acid chain elongation. This coordinated expression suggests the formation of metabolic channels or multi-enzyme complexes that enhance VLC-MUFA biosynthesis efficiency through substrate channeling and synchronized regulation. In marine copepods, for example, methyl-end desaturases and elongases are co-expressed and functionally diverse, enabling the efficient biosynthesis of physiologically essential fatty acids such as eicosapentaenoic acid and docosahexaenoic acid [47]. In cotton, the differential expression of *GhFAD2* and *GhFAD3* genes correlates with shifts in fatty acid composition during fiber development and stress responses, highlighting the dynamic transcriptional regulation of desaturase genes [48].

At the molecular level, the substrate specificity of FAD enzymes is determined by amino acid residues within the substrate-binding tunnel and the enzyme active site. Mutagenesis studies and molecular dynamics simulations have shown that even single amino acid changes can alter desaturase specificity by shifting the positioning of the substrate relative to the catalytic metal ions in the active site [49]. Structural studies of acyl carrier proteins (ACPs) and their interactions with desaturases further show that substrate chain length and conformation directly influence enzyme activity [50]. These insights provide a framework for understanding how *M. oleifera* FAD enzymes may have evolved substrate preferences for very long-chain saturated fatty acids, enabling the Δ15 desaturation reaction required for NA synthesis.

## 5. Transcriptional Regulatory Networks Governing Nervonic Acid Biosynthesis

MYB transcription factors are involved in multiple plant physiological and biochemical processes, including the regulation of lipid metabolism. In *Arabidopsis*, AtMYB30 has been shown to be involved in the synthesis of very-long-chain fatty acids (VLCFAs) [51]. In soybean, GmMYB331 can bind to the promoters of GmOLEO1/2/4 and activate their expression for oil accumulation [52]. In *Acer truncatum* Bunge, MYB and bZIP transcription factors were involved in regulating NA biosynthesis [53]. Moreover, the MYB96 transcription factor, a key regulator of fatty acid elongation, triggers cuticular wax accumulation in *Arabidopsis* leaves by directly binding to the promoters of *KCS1*, *KCS2*, *KCS6*, *β-KETOACYL REDUCTASE 1* (*KCR1*), and *ECERIFERUM 3* (*CER3*) genes [54]. This direct interaction drives the upregulation of enzymes essential for the chain elongation and desaturation steps for NA production.

**Figure 4:**
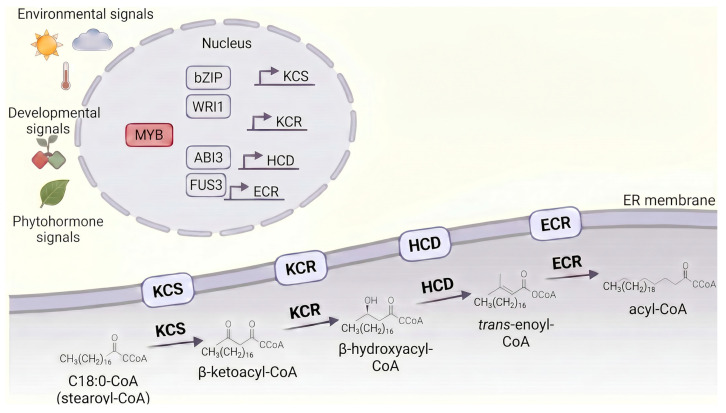
Regulatory model of nervonic acid biosynthesis pathway in Malania oleifera modulated by environmental, developmental, and phytohormone signals.

The regulatory influence of MYB transcription factors is further shaped by their integration into complex upstream signaling networks. These MYB factors are themselves regulated by hormonal cues including ABA and jasmonic acid [55,56], as well as bZIP proteins [53], forming multi-layered regulatory modules. Then, the MYB transcription factor modulates nervonic acid (NA) biosynthesis in response to both developmental signals and environmental stimuli (Figure 4). This multi-tiered regulatory architecture not only ensures precise spatial and temporal control of lipid biosynthesis, but also provides promising targets for metabolic engineering strategies aimed at increasing the NA content in *M. oleifera* and related crop species [57].

## 6. Multi-Omics Technologies for Decoding Nervonic Acid Biosynthetic Mechanisms

### 6.1. Transcriptomics Revealing Developmental Dynamics and Gene Co-Expression Networks

In *M. oleifera*, comprehensive RNA-Seq profiling of seeds at multiple developmental stages has facilitated the systematic identification of candidate genes associated with lipid metabolism and nervonic acid (NA) biosynthesis. The key enzymes such as fatty acid elongases, desaturases, and other components of the fatty acid biosynthetic pathway are critical for NA accumulation in seeds. Transcriptome analysis showed that *WRI1*, *ABI3*, and *FUS3* are involved in regulating the biosynthesis of NA-rich oils in *M. oleifera* seeds [58]. Moreover, in *Acer truncatum* seeds, WGCNA identified modules enriched with fatty acid desaturases and *KCS* genes whose expression levels closely correlated with NA accumulation, indicating their pivotal roles in the biosynthetic pathway [59]. These analyses not only highlight candidate genes, but also provide insights into the upstream regulatory networks and signaling pathways that shape NA biosynthesis.

Additionally, transcriptomic profiling of *M. oleifera* has revealed that host interactions and endogenous hormone signaling significantly alter the expression of genes related to nutrient synthesis and stress resistance, in turn influencing plant growth and metabolite accumulation [60]. These findings underscore the complexity of transcriptional regulation in NA biosynthesis, which integrates developmental cues, environmental stimuli, and even interspecies interactions (Figure 4).

### 6.2. Lipidomics and Metabolomics Depicting Dynamic Metabolite Profiles

Lipidomics, with its ability to precisely identify and quantify a vast range of lipid species from short-chain to ultra-long-chain fatty acids, is uniquely suited to track NA and its immediate precursors such as erucic acid. Advanced mass spectrometry techniques, typically coupled with chromatographic separation, enable the sensitive detection of these lipids, facilitating temporal profiling of their abundance throughout seed development and maturation. In *M. oleifera* seeds, a total of eleven compounds were identified from the fatty acid analysis, and nervonic acid was the most abundant fatty acid, followed by other major fatty acids including octadecenoic acid, docosenoic acid, dodecenoic acid, tetracosanoic acid, and others with relatively lower contents [61].

Integrating metabolomic data reveals how central metabolic pathways, including glycolysis, the tricarboxylic acid cycle, and amino acid metabolism, supply the precursors and cofactors essential for NA biosynthesis. The analysis identified 2964 lipid molecular species across 38 lipid subclasses, with TG (triglyceride) being the most prevalent, followed by DG (diglyceride), PG (phosphatidylglycerol), and Cer (ceramides) [15]. Studies in diverse plant systems have shown that fluctuations in sugar and amino acid pools directly influence lipid biosynthesis by altering energy availability and cellular redox balance [62,63]. In *M. oleifera* seeds, metabolomic profiling can identify key metabolites whose levels correlate with the onset and progression of NA accumulation, providing critical insights into the metabolic network that supports its synthesis. In addition, dynamic lipidomic and metabolomic profiling can capture temporal changes in metabolite pools, revealing the kinetics of NA biosynthesis and its coordination with seed developmental stages [64].

## 7. Metabolic Engineering Strategies and Challenges for Nervonic Acid Synthesis in *Malania oleifera*

### 7.1. Heterologous Overexpression of Key Rate-Limiting Enzyme Genes

Heterologous overexpression of the key rate-limiting enzyme genes in the NA biosynthetic pathway is a foundational strategy for both validating gene function and enhancing the production of this valuable very-long-chain monounsaturated fatty acid. In *M. oleifera*, genes encoding MoKCS and specific fatty acid desaturases (FADs) have been identified as the primary determinants of the elongation and desaturation steps in NA biosynthesis. Overexpressing these genes in *Arabidopsis thaliana* [65] and *Saccharomyces cerevisiae* [40] provides a direct approach to dissect their enzymatic functions and increase NA yield. Moreover, acyltransferase DGAT and GPAT from *M. oleifera* expressed in *Y. lipolytica* effectively improved the titer and content of nervonic acid [65]. FAD enzymes introduce double bonds within the acyl chain of fatty acids, determining the degree of unsaturation that is also critical to the biological activity and physical properties of NA-containing lipids [66].

The heterologous expression of enzymes for nervonic acid (NA) accumulation faces key challenges: an adequate supply of precursor erucic acid (C22:1) is essential to avoid metabolic bottlenecks, requiring co-expression strategies to enhance precursor biosynthesis, while the stoichiometric ratio of elongases and desaturases must be finely tuned to prevent intermediate buildup. Additionally, targeting heterologously expressed MoKCS and FAD enzymes to the endoplasmic reticulum (ER) (via signal peptides or transmembrane domains) and using strong tissue-specific promoters can improve catalytic efficiency and NA accumulation in desired tissues while minimizing unintended metabolic perturbations [67]. Furthermore, microbial platforms such as engineered yeast strains offer significant advantages, including rapid growth, ease of genetic manipulation, and the ability to perform eukaryotic post-translational modifications, making them attractive hosts for the industrial-scale production of NA [68].

### 7.2. Systematic Metabolic Engineering for Improved Nervonic Acid Production

*M. oleifera* has a unique endogenous ultra-long-chain fatty acid synthesis system with high KCS enzyme activity and natural preference for C24 fatty acid synthesis, which is an inherent advantage for high-yield NA engineering. *M. oleifera* accumulates oil exclusively in its seeds, enabling targeted and abundant enrichment of NA in seed tissues without compromising vegetative growth and normal plant development. Engineering optimization strategies are necessary to resolve the key limitations restricting industrial NA production in *M. oleifera*, including its lengthy growth cycle and low seed propagation efficiency, ambiguous regulatory mechanisms governing tissue-specific NA accumulation, and metabolic flux competition between NA and other ultra-long-chain fatty acids. In addition, systematic metabolic engineering represents a promising frontier for enhancing NA production efficiency, offering advantages over the conventional overexpression of individual structural genes. For example, in engineered oleaginous yeasts such as *Yarrowia lipolytica* and *Rhodosporidium toruloides*, the iterative expression of *KCS*, elongase (e.g., ELOVL6), and desaturase (e.g., MaOLE2) genes significantly increased the NA content, demonstrating the power of coordinated gene expression in metabolic engineering [36,69].

A critical challenge in the engineering of genes is achieving precise control over their expression levels and spatiotemporal patterns to avoid adverse effects on host growth and development. Overexpression of target genes can lead to metabolic imbalances, the accumulation of toxic intermediates, or the diversion of precursors from essential cellular processes, all of which can impair normal host physiology. Additionally, engineering strategies must incorporate promoters with tunable strength and inducible or tissue-specific expression systems to precisely modulate enzyme activity. Synthetic biology tools, such as CRISPR-based gene activation or repression systems and dynamic regulatory circuits, offer promising solutions to achieve this fine control. For example, the use of lipogenic phase-activated promoters in oleaginous yeasts has been shown to enhance NA accumulation specifically during the lipid biosynthesis phase, without compromising cell viability [69]. Similarly, engineering the ER structure regulator gene YlINO2 in *Y. lipolytica* increased lipid production by 39.3% without detrimental effects, illustrating the importance of balancing metabolic enhancement with cellular homeostasis [36].

These approaches, supported by advances in synthetic biology, metabolic engineering, and systems biology, hold great promise for developing high-yield NA-producing plants and microbial cell factories. However, the complexity of regulatory networks and the need to balance metabolic flux with cellular health remain significant challenges that require continued research and innovative engineering solutions.

### 7.3. Synthetic Biology and Construction of Microbial Cell Factories

The application of synthetic biology to reconstruct the complete NA biosynthetic pathway in microorganisms such as yeast represents a cutting-edge approach to the sustainable production of this valuable ultra-long-chain fatty acid. This strategy involves the modular assembly of distinct functional units within the microbial host, including precursor supply modules, elongation-desaturation modules, and transport-storage modules. This modular design allows for the flexible optimization and fine-tuning of each pathway segment, enabling efficient metabolic flux through the biosynthetic route and improved product titers [70]. Integrating KCS and FAD enzymes into microbial hosts, combined with systems biology-guided pathway balancing, has achieved significant improvements in NA yields, underscoring the importance of enzyme optimization in synthetic biology applications [71].

Despite recent advances, the microbial production of ultra-long-chain fatty acids such as nervonic acid (NA) still faces substantial challenges. A major limitation is the inherent cytotoxicity of these fatty acids, which damages membrane integrity and disrupts cellular homeostasis. To reduce toxicity, researchers have engineered transport and storage systems to sequester NA in lipid droplets or promote extracellular export. Additionally, metabolic flux imbalance in the reconstructed pathway often causes bottlenecks and intermediate accumulation due to unsynchronized precursor supply, elongation, and desaturation. Strategies including dynamic regulation, cofactor balancing, and alleviation of feedback inhibition have been adopted to optimize pathway efficiency. Industrial scale-up further requires the optimization of fermentation conditions, bioreactor design, and downstream processing. Adaptive laboratory evolution and high-throughput screening have facilitated the development of robust, stress-tolerant strains with stable NA production. Through the integration of synthetic biology, protein engineering, and process optimization, overcoming these hurdles will enable the cost-effective and sustainable industrial production of NA [72,73].

## 8. Prospects for Sustainable Utilization and Industrial Application

### 8.1. Synergistic Development of Artificial Cultivation and Ecological Protection

The sustainable development of *M. oleifera* cultivation requires a comprehensive understanding of the species’ reproductive biology, ecological conservation requirements, and innovative cultivation models that balance economic benefits with environmental protection. Studies on seed germination in wild plant species have demonstrated temperature-dependent germination requirements that reflect adaptation to local ecological conditions, insights that can inform artificial cultivation protocols to optimize seedling establishment and expand planting areas [74]. Furthermore, the identification of male-fertility genes and molecular markers in cultivated garlic has opened avenues for breeding programs aimed at overcoming reproductive barriers, enhancing reproductive success, and increasing genetic diversity in cultivated *M. oleifera* populations [75]. In situ conservation, through the establishment of protected areas in the native habitats of *M. oleifera*, is also vital for preserving wild populations and their genetic resources [76]. Additionally, tissue culture techniques have been successfully applied to related woody species, enabling the production of aseptic plantlets and stable regeneration, methods that can be adapted for the conservation and large-scale cultivation of *M. oleifera* [77]. These integrated conservation and cultivation strategies ensure the long-term survival of wild *M. oleifera* germplasm, while supporting the sustainable utilization of the species.

### 8.2. Development and Market Prospects of Nervonic Acid Products

Nervonic acid (NA), critical for brain development and neurological health, makes high-purity NA from *M. oleifera* seed oil a promising raw material for high-end nutritional supplements, functional foods, and medical nutrition, e.g., NA-fortified infant formulas may improve neurodevelopment in premature infants. Humans have limited endogenous NA synthesis, so dietary supplementation is essential; biotechnology advances (microbial fermentation, plant genetic engineering) have increased high-purity NA availability [78,79,80]. In pharmaceuticals, NA and its derivatives show potential as neuroprotective agents, supporting myelin sheath integrity and ameliorating neurodegenerative diseases. Animal studies demonstrate that NA improves cognitive function, reduces neuroinflammation, and protects against oxidative stress; NA-containing lipid formulations have been explored for targeted nervous system drug delivery [12,81,82]. Cosmetically, NA’s skin barrier repair and moisturizing abilities, along with antioxidant/anti-inflammatory properties, make it valuable for high-end skincare, aligning with demand for natural ingredients [80,83]. Commercially, NA viability depends on addressing raw material, extraction, and purity challenges. Enzymatic enrichment achieves over 97% purity, while the metabolic engineering of oleaginous yeasts enables sustainable, high-yield production [84,85,86]. Market interest grows due to neurological disorders, aging populations, and the demand for natural functional ingredients; regulatory evaluations will facilitate broader NA product acceptance, supported by biosynthetic/extraction advances and evidence of its health benefits [78,79,80].

## 9. Conclusions

The extraordinarily high NA accumulation in *M. oleifera* is attributed to a compartmentalized metabolic system, though our mechanistic understanding remains partially conceptual. Current models covering plastid synthesis, ER elongation, Δ15 desaturation, and the functional divergence of KCS and FAD families are largely based on homology inference, while the rate-limiting function of KCS, the structure-determining role of Δ15 desaturase, and the regulatory networks of key transcription factors (MYB, bZIP, WRI1, etc.) lack rigorous in vivo validation in *M. oleifera*. Unconfirmed subcellular localization and variable catalytic properties of specific enzyme isoforms further leave the metabolic flux partitioning between NA synthesis and competing ultra-long-chain fatty acid pathways poorly defined.

This review provides a dedicated summary of the core bottlenecks restricting industrial-scale NA production. Two major intrinsic constraints limit practical biotechnological application: metabolic flux imbalance and insufficient precursor supply. In *M. oleifera*, parallel ultra-long-chain fatty acid branch pathways compete for metabolic resources and disperse synthetic flux, thereby suppressing NA accumulation and limiting the final product yield. Meanwhile, the limited endogenous pool of C18:0 oleic acid precursors constrains continuous acyl-chain elongation and downstream NA biosynthesis, representing another key rate-limiting factor for industrial production. These unresolved metabolic defects are the primary cause of suboptimal NA yields in current heterologous expression systems.

To advance from descriptive homology-based models to rational and predictable metabolic engineering, future studies must focus on the empirical validation of enzyme localization, the systematic identification of upstream regulators, and the quantitative characterization of metabolic flux dynamics. Technically, flux redirection engineering to eliminate competitive branch pathways and targeted precursor pool enhancement to enrich C18:0 substrates will be essential to break through current industrial limitations. By critically integrating verified mechanisms, unresolved knowledge gaps, and industrial bottlenecks, this review provides a comprehensive framework to guide subsequent basic research and accelerate the sustainable industrialization of this high-value bioactive resource.

## Data Availability

No new data were created or analyzed in this study. Data sharing is not applicable to this article.
